# Minimum Cost Estimation of a Baseline Survey for a Molecular Epidemiology Cohort Study: Collecting Participants in a Model Region in Japan

**DOI:** 10.2188/jea.JE20150163

**Published:** 2016-10-05

**Authors:** Izumi Mishiro, Norie Sawada, Motoki Iwasaki, Kayo Ohashi, Shoichiro Tsugane

**Affiliations:** Epidemiology and Prevention Group, Research Center for Cancer Prevention and Screening, National Cancer Center, Tokyo, Japan

**Keywords:** molecular epidemiology study, research fund, cost, regional study office

## Abstract

**Background:**

Some recent molecular epidemiology studies of the effects of genetic and environmental factors on human health have required the enrollment of more than 100 000 participants and the involvement of regional study offices across the country. Although regional study office investigators play a critical role in these studies, including the acquisition of funds, this role is rarely discussed.

**Methods:**

We first differentiated the functions of the regional and central study offices. We then investigated the minimum number of items required and approximate cost of a molecular epidemiology study enrolling 7400 participants from a model region with a population of 100 000 for a 4-year baseline survey using a standard protocol developed based on the protocol of Japan Public Health Center-based Prospective Study for the Next Generation.

**Results:**

The functions of the regional study office were identified, and individual expenses were itemized. The total cost of the 4-year baseline survey was 153 million yen, excluding consumption tax. Accounting difficulties in conducting the survey were clarified.

**Conclusions:**

We investigated a standardized example of the tasks and total actual costs of a regional study office. Our approach is easy to utilize and will help improve the management of regional study offices in future molecular epidemiology studies.

## INTRODUCTION

Large-scale population-based molecular epidemiology studies aimed at elucidating the effects of genetic and environmental factors on human health have recently been initiated. The majority of studies are conducted as multisite consortia of regional study offices, which are responsible for the recruitment, collection, and follow-up of participants in the surrounding geographical area.^1^

Although cohort study profiles have been widely reported, little information is available on the accounting aspects of these studies. We therefore analyzed accounting data from an existing study, which was performed as a pilot study aimed at developing an example (standard) protocol for subsequent molecular epidemiology cohort studies in Japan.^2^ This standard protocol was developed as part of a project of government-commissioned research carried out by research members of the Japan Public Health Center-based Prospective Study for the Next Generation (JPHC-NEXT) and was based on their own protocol for an ongoing cohort study supported by the National Cancer Center Research and Development Fund.^3^ The improved standard protocol method was particularly focused on molecular epidemiology and includes the full-genome sequencing of all samples in the future with the informed consent of participants under the Ethical Guidelines for Human Genome/Gene Analysis Research, as fully revised in 2013.^4^ The standard protocol was released to the public in March 2014.^5^ The outline is shown in eAppendix 1.

Figure shows an overview of the standard protocol cohort study. Following the collection of written informed consent from volunteers, the design included a baseline survey with a self-administered questionnaire on lifestyle behaviors, collection of urine and blood to store and extract genomic DNA, and health checkup data; a 20-year follow-up system for mortality, migration, and incidence of cancer and other lifestyle diseases; and an additional survey to be performed every 5 years. Collected samples and data were to be forwarded to the central study office to ensure uniform storage and maintenance for future research use and sharing under the supervision of the institutional review board (IRB).

**Figure.**
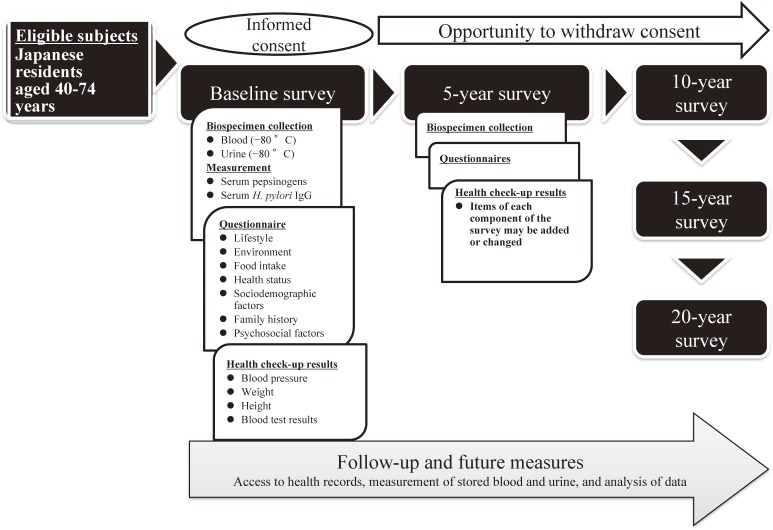
Components of surveys in a cohort study according to the standard protocol. Study components include the baseline, which includes the collection of biospecimens and measurements, questionnaires, and health check-up results. The follow-up and biorepository period will be 20 years from enrollment. Every 5 years from the baseline survey, the same type of cross-sectional survey will be performed. Survey items may be added or revised according to medical developments that occur following the baseline survey.

In this study, we applied the standard protocol to a model region with a population of 100 000. We evaluated the minimum number of required items and calculated the approximate expenses involved in enrolling participants at municipal health check-ups in a model region during a baseline survey according to the standard protocol. This information is easy to utilize and will help facilitate the management of future studies in new regions.

## METHODS

### Estimation of participants

The population of the model region of the standard protocol was assumed to be 100 000.^5^ The standard protocol requires the inclusion of all residents aged 40 to 74 years and the tailoring of the sample collection strategy to meet specific regional circumstances.

The Vital Statistics of Japan 2013 indicate a total population of 125 million, of whom 47% were aged 40 to 74 years.^6^ Therefore, a total of 46 868 residents of the model region were potentially eligible for the study.

Blood and urine samples for molecular epidemiology studies can be collected at health check-up facilities. Here, we focused on National Health Insurance members who might take municipal health check-ups, to ensure our method is suitable for any region in Japan.

In Japan, all citizens are required by law to be covered by either employee-based or community-based healthcare insurance. National Health Insurance by municipalities is available for those who are not conventionally employed, such as farmers and self-employed and retired persons. In 2008, special health check-ups (Tokutei Kenshin) and guidance by healthcare insurers, with a focus on metabolic syndrome, were mandated for those of 40–74 years of age. As a health insurer, municipalities provide general check-ups and special health check-ups for insured persons and their dependents (municipal health check-ups). Municipalities also provide nationwide screening programs for cancer of the stomach, uterus, breast, lung, and colorectum. We therefore expected that some of our study subjects would undergo special health check-ups and/or cancer screening at municipal health check-up sites.

We first estimated the population of each age group in the model region, as well as the numbers of National Health Insurance members, municipal health check-up examinees, and study participants (Table 1).^7^^,^^8^ The average reported participation rate in municipal health check-ups across all prefectures in Japan is 45%,^7^ which was applied to the model region. The expected participation rate in the present study was 80%, based on our pilot study results,^9^ and the estimated number of enrollees was 7414.

**Table 1.** Estimation of populations, National Health Insurance members, special health check-up examinees, and study participants in a model region by age group

Age group, years	Population			National Health Insurance members			
	
	Japan		Model region	Participation in Japan^b^	Model region		
		
	Number^a^ (× 1000)	Proportion/whole population	Number		Number^c^	Municipal health check-up examinees^d^	Study participants^e^
All	125 704	100.000%	100 000				
40–44	9517	7.571%	7571	23.9%	1809	814	651
45–49	8279	6.586%	6586	24.3%	1600	720	576
50–54	7637	6.075%	6075	25.5%	1549	697	558
55–59	7658	6.092%	6092	31.6%	1925	866	693
60–64	9608	7.643%	7643	51.8%	3959	1782	1425
65–69	8654	6.884%	6884	71.9%	4950	2227	1782
70–74	7562	6.016%	6016	79.8%	4801	2160	1728
40–74	58 915	46.868%	46 868		20 594	9267	7414
				(Number per year)	(5150)	(2320)	(1850)

^a^Japanese population on 1 October 2013 by age.^5^

^b^Data on the situation of specific health checkups for National Health Insurance members.^7^

^c^(Model region population) × (participation in National Health Insurance in Japan).

^d^Participation rate of 45% of National Health Insurance members in a model region.

^e^Participation rate of 80% of municipal health check-up examinees.

### Schedule

The model region was divided into four sections of almost equal population size, so that each section would be required to enroll 1853 volunteers per year. This would, in turn, require inviting approximately 5200 individuals to 30 days of health check-ups per year in order to conduct 2400 examinations; 7400 volunteers would be enrolled over 4 years. Following the baseline survey, the second survey is scheduled to begin in the 6th year of the study. This schedule is intended to help maintain the continuous and uniform function of the regional study office.

### Identifying functions of regional study office

Table 2 lists works considered essential for the study according to the standard protocol. The central study office is responsible for maintaining the whole cohort study; specifying methods, tools, and forms, ensuring the safe management of sensitive information; and maintaining the quality of surveys and of the biorepository, sample storage, and data analysis. The central study office is also responsible for other related work, including ethical, legal, and social issues, as well as the advertising strategy during long study periods; establishing councils; planning the future of the study; and providing appropriate support for regional study offices.^9^ Investigators in the regional study office should focus on completion of the baseline survey and follow-up registration.

**Table 2.** Work process and role of study offices in the standard protocol

Work Phase/Process		Role of offices	

		Central	Regional
Planning Phase			
1.	Development of standard operating protocols, study materials, methods for data and sample collection with safety management manual	Main	
2.	Design of survey details using a site-specific strategy	Main	Main
3.	Submitting institutional review board applications	Main	Main
4.	Scheduling, negotiating, and contracting with local government offices and related organizations	Main	Main
5.	Decision-making for survey manual details	Support	Main
6.	Securing staff resources, education, and training	Support	Main
7.	Procuring and managing study materials	Support	Main
8.	Regional advertising for the study	Support	Main
Conduct Phase			
1.	Distributing questionnaires		Main
2.	Personnel distribution, transportation and accommodation, and material delivery		Main
3.	Recruiting and enrolling participants and asking for informed consent at health check-up sites	Support	Main
4.	Collecting blood and urine samples; blood centrifuging and dispensing	Support	Main
5.	Collecting questionnaires from participants and providing gift cards	Support	Main
6.	Delivering defined results to participants	Support	Main
Checking and Follow-up Phase			
1.	Organizing data collection, cleaning, and filing in the region	Support	Main
2.	Short-term storage of blood samples at −80°C		Main
3.	Responding, recording, and reporting problems and cases	Support	Main
4.	Sending collected study materials and data to the central study office	Support	Main
**5.**	Improving operation procedures; revising the regional survey manual	Support	Main
6.	Follow-up and updating participant records, lodging them at the central study office	Support	Main
7.	Sharing study results with local government and conducting outreach projects, such as lectures, to communicate details of the study	Support	Main
8.	Distribution of a newsletter and inquiry response	Main	Support
9.	Developing a system and method for analyzing anonymous information	Main	
**10.**	Storing final study materials and data; construction and management of a biorepository and study database	Main	
11.	Handling complaints and withdrawal of consent	Main	Support
12.	Return of the study results to society	Main	Support

In the planning phase, investigators in the regional study office should prepare a site-specific research protocol and informed consent form for submission to the IRB. They should also be involved in the enrollment of participants at the municipal health check-up sites in cooperation with local municipal governments.

The conduct phase includes tasks regarding the collection of data and samples and the return of results. Investigators should ask volunteers for written informed consent and collect questionnaires and biospecimens. The standard protocol indicates that the following test results should be returned to individual participants: advice regarding dietary excesses or deficiencies; risk of cancer or cardiovascular disease in the next 10 years, based on assessment of lifestyle; risk of gastric cancer, based on serum levels of *Helicobacter pylori* antibody and chronic gastritis, based on pepsinogen levels. As a further incentive to complete and return the questionnaire, enrollees should also be provided with a 1000 yen gift card. All biospecimens should be temporarily stored at the regional study office and then sent to the biorepository maintained by the central study office.

An important step in the evaluation phase is data organization, in which data is cleaned and filed and data and collected samples are delivered to the central study office. Follow-up of subjects providing informed consent should start within a year and include the retrieval of incident cases from major hospitals in the region or the cancer registry system and the relocation or death of subjects from resident records of the municipality. The cause of death should be registered based on the Vital Statistics of Japan, with permission of the Ministry of Health, Labour and Welfare.

### Estimation of total cost

Based on this strategy, we then estimated the necessary expenses of performing the functions of a regional study office using the annual budget system, which requires the annual budget to be spent within the fiscal year to which it is applied.

The regional study office was expected to already possess basic tools and infrastructure for office and laboratory work. We also excluded costs associated with regular employees acting in at least three investigator roles (typically the principal investigator, site manager, and data and laboratory manager) and a secretary role. Unit prices were primarily obtained from accounting reports of a pilot study for the standard protocol. After the minimum numbers of each item were decided, unit prices were adjusted in accordance with the number of ordered commodities based on the supplier price estimate. Unit costs for daily employees and meeting expenses were obtained using administrative guidance for public research expenses from the National Cancer Center.

Miscellaneous expenses for supplies and materials, postage, and outsourcing are expected. Extra monthly costs were approximated and included as ‘others’. Materials should be ordered with a 20% margin on the estimated number.

## RESULTS

### Estimation of total cost

Table 3 shows an itemized summary of annual expenses. Initial expenses of a regional study office were estimated and totaled using annual budgets between the 1st and 4th fiscal years. The total expense after 4 years was calculated to be 153 million yen, consisting of approximately 41 million yen in the first year and 37.4 million yen per year for the 2nd to 4th years. These figures do not include consumption tax.

**Table 3.** Cost estimation in a regional study office

Item	Unit Price (yen)	1st year		2nd to 4th year		Total (yen)
	
		Number	(yen)	Number	(yen)	
**Salaries and wages**						
**Full-time employees by yearly contract**						
Researcher	3 900 000	1	3 900 000	1	3 900 000	15 600 000
Coordinator	3 200 000	1	3 200 000	1	3 200 000	12 800 000
**Part-time employees for data entry at a regional study office (9:00–12:00)**						
Research assistant	944 000	1	944 000	1	944 000	3 776 000
**Daily employees at health check-up sites, 30 days/year**						
Nurses (*n* = 5)	7800	150	1 170 000	150	1 170 000	4 680 000
Others (*n* = 15)	6900	450	3 105 000	450	3 105 000	12 420 000
**Dispensing and laboratory work, daily, 30 days/year**						
Assistants (*n* = 2)	7800	60	468 000	60	468 000	1 872 000
**Follow-up survey, daily, 3 days/week, 20 weeks/year**						
Assistants (*n* = 3)	6900	180	1 242 000	180	1 242 000	4 968 000
**Sub Total**			**14 029 000**		**14 029 000**	**56 116 000**
**Travel**						
Health check-up sites (*n* = 25, 30 days)	3000	750	2 250 000	750	2 250 000	9 000 000
Central study office (*n* = 4, 4 times)	100 000	16	1 600 000	16	1 600 000	6 400 000
**Sub Total**			**3 850 000**		**3 850 000**	**15 400 000**
**Equipment**						
Deep freezer (−80°C, 700 L)	3 000 000	1	3 000 000	0	0	3 000 000
Computer	250 000	2	500 000	0	0	500 000
**Sub Total**			**3 500 000**		**0**	**3 500 000**
**Supplies and materials**						
Sample collection	674	2800	1 887 200	2800	1 887 200	7 548 800
Writing utensils	100	2800	280 000	2800	280 000	1 120 000
1000-yen gift cards	1040	2300	2 392 000	2300	2 392 000	9 568 000
Other (monthly)	50 000	12	600 000	12	600 000	2 400 000
**Sub Total**			**5 159 200**		**5 159 200**	**20 636 800**
**Others**						
**Printing**						
OCR-capable questionnaires	293	6200	1 816 600	6200	1 816 600	7 266 400
Information booklets	45	6200	279 000	6200	279 000	1 116 000
Invitation leaflets	15	6200	93 000	6200	93 000	372 000
Envelopes (large)	39	6200	241 800	6200	241 800	967 200
Envelopes (small)	28	6200	173 600	6200	173 600	694 400
Informed consent form	19	2800	53 200	2800	53 200	212 800
Sampling sheet	19	2800	53 200	2800	53 200	212 800
**Outsourcing**						
Data processing for listing and labeling	200	6200	1 240 000	6200	1 240 000	4 960 000
Data processing for questionnaire	795	2300	1 828 500	2300	1 828 500	7 314 000
Biological assays	1400	2300	3 220 000	2300	3 220 000	12 880 000
Advertisement	600 000	1	600 000	1	600 000	2 400 000
Document enclosure and shipping	94	6200	582 800	6200	582 800	2 331 200
Other (monthly)	100 000	12	1 200 000	12	1 200 000	4 800 000
**Postage**						
Questionnaire	250	6200	1 550 000	6200	1 550 000	6 200 000
Returning results	82	4600	377 200	4600	377 200	1 508 800
Other (monthly)	80 000	12	960 000	12	960 000	3 840 000
**Meeting**						
Meeting for training	2000	50	100 000	50	100 000	400 000
**Sub Total**			**14 368 900**		**14 368 900**	**57 475 600**
**GRAND TOTAL**			**40 907 100**		**37 407 100**	**153 128 400**

Consumption tax is not included.

### Salaries and wages

A post-doctoral researcher with an hourly base rate of 1610 yen and coordinator of 1300 yen are required at the regional study office as full-time employees (1952 h per year). Their role is to assist investigators from the planning phase, oversee the production of study materials and scheduling of daily employees, and organize sites and a laboratory to perform the baseline survey. Total annual cost for these employees was calculated to be 7.1 million yen.

A research assistant is required to enter data and handle personal data at the regional study office 3 hours per day throughout the year. Total annual salary cost for this part-time employee was calculated to be 0.9 million yen.

In the conduct phase, municipal health check-up sites receiving more than 100 eligible persons per day require many part-time employees. To enroll 80 participants, the following employee numbers are required: registration desk, 2; guide, 1; explanation of the study in the waiting room, 1; informed consent booths, 5; urine collection, 2; blood collection, 2; questionnaire collection, 5; and provision of a gift card upon completion of the questionnaire, 2. Arrangements should be such that the flow of the health check-up is not disrupted. A period of 30 to 60 min is required for a participant to fill out the baseline questionnaires used for the standard protocol. The questionnaire should be delivered by mail in advance with a letter inviting subjects to bring the completed questionnaire to the study. To collect completed questionnaires on the check-up day, assistants are required to attend to participants.

According to the plan, each site requires a total of 20 part-time employees (5 nurses and 15 graduate school students) under a daily contract. This health check-up recruitment program should be implemented 30 times per year in the model region. Employees should be allocated based on their qualifications. Two laboratory assistants are also required to process biospecimens.

In the evaluation and follow-up phase, three research assistants are required to register follow-up information for 60 days per year (3 days per week, 20 weeks per year). The total annual cost for daily employees was calculated to be 6.0 million yen. The subtotal of salaries and wages was calculated to be 14.0 million yen.

### Travel expenses

A total of 25 people are expected to require single-day travel to nearby districts for 30 days per year. Further, regional study office staff expenses were calculated to include four employees taking overnight business trips to the central study office to attend meetings four times per year. The subtotal of travel expenses was calculated to be 3.9 million yen per year.

### Equipment, supplies, and materials

A deep freezer (−80°C, 700 L capacity horizontal freezer with a double cooling system) for the temporary storage of samples and two computers to store personal information offline are required from the first year. The subtotal of equipment expenses was calculated to be 3.5 million yen.

Estimates for supplies and materials include experimental tools for blood and urine collection, dispensing, and storage. Thousand-yen gift cards for study participants who completed questionnaires were also included. The subtotal of supplies and materials was calculated to be 5.2 million yen per year.

### Printing, outsourcing, and others

The study questionnaire, information booklet, advertising leaflet, and invitation letter should be enclosed in an envelope. For documents sent to residents or participants, it is preferable that personal information be written only once, indicating the need for windowed envelopes. Informed consent and blood sampling register forms should be prepared on two sheets of carbonless copy paper.

Outsourcing is used for fixed types of work, such as data processing, document enclosure, and shipping. Commercial laboratories are considered suitable for serum pepsinogen and *Helicobacter pylori* IgG testing. Local advertising agencies tend to have better personal networks established within their region and be capable of more effective outreach activities than national advertising agencies.

The subtotal of other expenses, including printing, outsourcing, postage, and meetings, was calculated to be 14.4 million yen per year.

## DISCUSSION

In this study, we investigated the minimum number of items required and approximate cost of enrolling 7400 participants in a model region with a population of 100 000 for a 4-year baseline epidemiological survey using a standard protocol. This protocol was designed to promote molecular cohort studies via the integration of genomic data and electronic medical records.^5^ Here, we investigated the feasibility of applying our approach to the baseline survey of a molecular epidemiology cohort study according to a standard protocol. The total cost of this model survey was 153 million yen. Our approach is easy to utilize and will help improve the management of regional study offices in future molecular epidemiology studies.

We used a simplified version of a specific study protocol and study subjects for our estimation. Investigators should try to as many study participants as possible. National Health Insurance by municipalities covers about 50 million subscribers in Japan, 64% of whom are over 60 years of age.^8^^,^^10^ Therefore, our method has the disadvantage of enrolling fewer participants aged 40 to 59 years.^9^ An additional strategy may be to invite eligible subjects and recruit participants at regional health examination centers, which are attended by members of not only the National Health Insurance scheme but also the Social Insurance scheme. For each health examination center, this would increase the payroll for part-time employees by 3 million yen.

Second, as a proportion of residents do not attend annual health check-ups, elaborate methods to invite candidates who do not attend annual health check-ups are required. Although we estimated the participation rate in the check-ups to be 45%, it was around 35% in the regions of our pilot study. To ensure a sufficient number of study participants, we should have repeated recruitment in the same district the following year, which would involve additional expense.

Higher participation rates in health check-ups achieved by each district allow higher percentages of the population to be covered by the cohort study. Effective but ethically fair municipality- or local area-based advertising methods are required to motivate eligible residents to participate in both the health check-up and the study. Moreover, municipal governments joining the research project would likely experience the secondary benefit of increased involvement in annual health check-ups and increased participation in health promotion efforts in their region.

The costs of this study are designed to be as low as possible, so that investigators may add requirements on a case-by-case basis. For example, salaries for daily employees at municipal health check-up sites were obtained from the accounting requirements of the Health and Labour Sciences Research Grants scheme of the Ministry of Health, Labour and Welfare, which show a unit price for a skilled worker of 7800 yen per day.^11^ This cost doubles when nurses or research assistants are sent from a temporary recruitment agency. Regarding the requirements of Grants-in-Aid for Scientific Research by the Ministry of Education, Culture, Sports, Science and Technology, wages are decided more flexibly based on institutional regulations^12^; publicly available regulations (accessible via websites) indicate a maximum hourly wage for post-doctoral fellows and skilled technicians of approximately 1500 yen, and a mean hourly wage for short-term employment with the Japanese Nursing Association website of 1873 yen.^13^ Overall, the relatively low daily wages stipulated for short-term employees under current research grant regulations will make it difficult for investigators to secure sufficient personnel resources. For example, a regional office of our pilot study failed to hire a researcher under a yearly contract, and the principal investigator should have devoted much more time and effort than was originally planned.

It was also difficult for the region to recruit sufficient numbers of daily employees at the health check-up sites. The investigator subsequently distributed the questionnaire book and an envelope after obtaining informed consent and asked participants to complete it at home and return it by mail within 1 or 2 weeks. This plan would reduce the number of daily employees required to collect questionnaires (*n* = 5) and to provide gift cards in return for completing the questionnaire (*n* = 2). This would reduce the time required of participants, but would also reduce the response rate and increase the subsequent workload of investigators.

Staff working in informed consent booths require sufficient knowledge of the molecular epidemiology study, so qualified doctors and nurses obliged to confidentiality are preferred, although not essential; a trained research coordinator with a contract containing an obligation to confidentiality may also perform this duty. We therefore included the cost of a 2-day training program at the regional study office to train 25 daily employees of the municipal health check-up facilities to perform this function. This training may use course material provided by the Genomics and Research Coordinator Program of the Japan Society of Human Genetics.^14^ Initial certification requires attendance at a 1-day program, which is usually held in Tokyo and consists of five lectures and a test. When travel costs are included, this certification may be expensive.

According to the standard protocol, the central study office should cover follow-up registration throughout the country. We therefore included the same wages for data entry without change for 4 years. Additional non-personnel costs arising from the follow-up survey should also be included, such as medical data processing fees.

In addition, travel expenses were estimated based on a single-day trip from the regional study office to each health check-up site in the district. When health check-ups start early in the morning, a proportion of employees might be required to stay near the site over the preceding night and thus incur additional travel expenses. In addition, unexpected problems with accessibility to the health check-up sites might arise, which would increase the cost. Investigators in a regional office of our pilot study in the west of Japan had their research field in a distant location in the east, causing travel expenses to be far higher than in the present estimation.

In addition, for equipment and materials, we reduced initial costs by excluding basic laboratory equipment, such as centrifuges. The total study cost will vary widely depending on the facilities required. For instance, the cost would increase in cases requiring flooring reinforcement and electric construction for the installation of a deep freezer.

Printing and material fees vary depending on the quantity ordered. The cost may decrease in cases where all printed matter for the 4 years could be obtained as a single order. An annual order might be a better approach for cases where printing requires revision. Expiration dates should be considered.

Although we estimated the minimum cost of advertising, continuous communication in the region is essential to procure and retain the trust of study participants. Costs related to providing information through lectures, community notices, and radio or cable television programs were included. More effective methods and attractive contents should be developed at appropriate price points to increase the number of study participants and decrease withdrawals.

Among other issues, dividing the work done for a health check-up provided by a municipality from that done for a research project is especially challenging. Investigators may find it difficult to manage cooperation with local governments. Although health check-ups and research projects share similar health promotion aims, the work done cannot be shared equally and should instead be divided by cost. Regional investigators should carefully discuss the procedure for the conduct of the survey with the respective municipalities to reach consensus. In fact, cooperation with local governments and the need for long-term continuity may not be amenable to methods of funding using short-term competitive research funds. A new funding framework for longitudinal prospective studies with population-based surveys is required.

The functions of a regional study office should be identified according to the model of the study consortium to which it belongs. We did not discuss the costs of the central study office in this study. Given that the costs of a central study office may vary widely depending on the concept of the consortium, further investigation into the minimum cost estimation of the central study office is warranted. A baseline survey for a total of 100 000 participants from all over the country is a large-scale research enterprise, and the follow-up survey can take 20 years or more. Once started, investigators of the central study office would face the difficulty of acquiring funding for more than 20 years to allow the collected data and samples to be utilized. Considering that the project is in the national or public interest, it should be implemented via long-term funding rather than short-term competitive funding.

The findings of this report will help facilitate the acquisition of research funds, problem solving, and the promotion of molecular epidemiology studies in Japan.

## ONLINE ONLY MATERIAL

eAppendix 1. Outline of the standard protocol.
