# Supplementary material for: Minimum Cost Estimation of a Baseline Survey for a Molecular Epidemiology Cohort Study: Collecting Participants in a Model Region in Japan
Source: J Epidemiol. 2016 Oct 5;26(10):522–9. doi: 10.2188/jea.JE20150163 (PMC5037249; doi:10.2188/jea.JE20150163)
Supplement: eAppendix 1. [file je-26-522-s001.pdf]

eAppendix 1. Outline of the standard protocol

From: The standard protocol, Structuring of a large-scale molecular epidemiology cohort study. 1.30.2014 3rd edition

(Financed and authorized by the Japanese Ministry of Education, Culture, Sports, Science and Technology)

Outline of the study protocol

The objectives of this molecular epidemiology cohort study are to maintain and promote the nation's health; in particular, to elucidate the nature and cause of chronic noncommunicable diseases, such as cancer, and to provide evidence for the prevention, control, and treatment of such diseases with regard to environmental and genetic factors and their interaction.

Specifically, we will conduct a baseline survey of Japanese residents between the ages of 40 and 74 years living in the target area, with informed consent. We will collect information through a self-administered lifestyle questionnaire and will use data from the participants' health check-ups. Blood and urine samples will be collected to store and investigate biological information, including genetic information like single nucleotide

polymorphisms (SNPs) in genes. The survey is scheduled to be repeated every 5 years. The study participants will be followed for mortality, migration, and incidence of cancer, cardiovascular disease, and other major diseases. We will construct a substantial study infrastructure through long-term and highly sensitive follow-up registrations to assist researchers with each study analysis using the stored samples and biological data as soon as possible.

1.1 Targeted area

___________area (population area of 100,000 people will be the standard)

1.2 Number of prospective participants

____

1.3 Definition of study subjects

Men and women within the age range of 40-74 years living in the targeted area with resident registration and who have provided informed consent to participate in the study.

1.4 Study period: (Registration period) Study approval date to ______ year, end of December; (follow-up period) 20 years from baseline registration; (analysis period) 10 years from termination of follow-up.

1.5 Baseline Survey: A self-administered lifestyle questionnaire, data from participant health check-ups, a 7-mL blood sample provided by study participants during their health check-up (plasma, white blood cell DNA, red blood cells), 1.5 mL of serum remaining after routine check-up (if blood collection is not scheduled in the check-up, then additional blood for serum will be drawn, and a fixed quantity of the blood collected for the health check-up will be used and referred to as additional blood drawn), and 4 mL of urine.

1.6 Method

- Study subjects will be identified based on the residence registry of municipalities in the study area.
- From the study subjects, we will collect blood and urine samples and health check-up data focusing on those who received special check-ups, detailed health check-ups, and cancer check-ups. We will also set up a site for providing blood and urine samples for

those not receiving a health check-up, and request participation in the study.

- Total nutrient intake, estimated risk of developing cancer or cardiovascular disease in the next 10 years, and results of examination for serum *Helicobacter pylori* antibody and serum pepsinogen will be calculated based on the collected information and returned to the participants.

Follow-up study will be conducted concerning mortality, migration, incidence of cancer and other lifestyle diseases (stroke, myocardial infarction, sudden death, congestive heart failure, aortic aneurysm rupture, aortic dissection), onset of psychiatric disease (e.g., depression, dementia), and self-reported disease (e.g., diabetes, fracture, ophthalmopathy). Samples associated with health insurance information and medical examination (long-term nursing insurance, details of medical expenses, examination samples, biological specimens, and information regarding preserved samples, such as extirpated samples) will be collected. We plan to actively use preexisting computerized medical information.
